# Association between serum bone biomarker levels and therapeutic response to abatacept in patients with rheumatoid arthritis (RA): a multicenter, prospective, and observational RA ultrasound cohort study in Japan

**DOI:** 10.1186/s12891-021-04392-5

**Published:** 2021-06-01

**Authors:** Shin-ya Kawashiri, Yushiro Endo, Ayako Nishino, Momoko Okamoto, Sosuke Tsuji, Ayuko Takatani, Toshimasa Shimizu, Remi Sumiyoshi, Tomohiro Koga, Naoki Iwamoto, Kunihiro Ichinose, Mami Tamai, Hideki Nakamura, Tomoki Origuchi, Toshiyuki Aramaki, Yukitaka Ueki, Tamami Yoshitama, Nobutaka Eiraku, Naoki Matsuoka, Akitomo Okada, Keita Fujikawa, Hiroaki Hamada, Shuji Nagano, Yoshifumi Tada, Atsushi Kawakami

**Affiliations:** 1 Departments of Community Medicine, Nagasaki University Graduate School of Medical Sciences, 1-12-4 Sakamoto, Nagasaki, 852-8523 Japan; 2 Departments of Immunology and Rheumatology, Division of Advanced Preventive Medical Sciences, Nagasaki University Graduate School of Medical Sciences, Nagasaki, Japan; 3 Kyushu Multicenter Rheumatoid Arthritis Ultrasound Prospective Observational Cohort Study (KUDOS) Group, Kyushu, Japan

**Keywords:** Rheumatoid arthritis, Musculoskeletal ultrasound, Abatacept, Dickkopf-1, Osteoprotegerin, Power Doppler, Sclerostin

## Abstract

**Background:**

To evaluate the effect of treatment on serum bone biomarkers and explore whether serum bone biomarkers are associated with therapeutic response in rheumatoid arthritis (RA) patients treated with abatacept.

**Methods:**

We enrolled 59 RA patients treated with abatacept from a multicenter, exploratory, short-term, prospective and observational ultrasound cohort study of patients who received biologic or targeted synthetic disease-modifying antirheumatic drug (DMARD) therapy. We evaluated the patients’ clinical disease activity and musculoskeletal ultrasound (MSUS) scores. The serum concentrations of five bone biomarkers were evaluated (dickkopf-1 [Dkk-1], sclerostin [SOST], osteocalcin [OC], osteopontin [OPN], and osteoprotegerin [OPG]) by multiplex bead assays at baseline, 3, and 6 months: the change over 6 months was defined as the Δ value. ‘Power Doppler (PD) responder’ was defined as a patient whose Δtotal PD score over 6 months was greater than the median change.

**Results:**

Abatacept significantly improved the clinical disease activity and MSUS score over 6 months. Serum OPG was significantly elevated at 6 months after the abatacept introduction (*p* = 0.016). The ΔSOST and ΔOPG were significantly greater in the PD responders versus the non-PD responders (*p* = 0.0041 and 0.0073, respectively). The serum Dkk-1 at baseline was significantly lower in the PD responders (*n* = 30) vs. the non-PD responders (*n* = 29) (*p* = 0.026). A multivariate logistic regression analysis showed that the serum Dkk-1 at baseline (odds ratio 0.50, 95% confidence interval [CI] 0.23–0.91, *p* = 0.043) was an independent predictor of PD responder status.

**Conclusion:**

Serum levels of bone biomarkers may be useful for predicting RA patients’ therapeutic responses to abatacept.

**Trial registration:**

Name of the registry: Assessment of therapeutic responsiveness by imaging of the joints in patients with rheumatoid arthritis; A observational cohort study

Trial registration number: UMIN000012524

Date of registration: 12/9/2013

URL of trial registry record: https://upload.umin.ac.jp/cgi-open-bin/ctr/ctr_view.cgi?recptno=R000014657

**Supplementary Information:**

The online version contains supplementary material available at 10.1186/s12891-021-04392-5.

## Background

Rheumatoid arthritis (RA) is a chronic inflammatory joint disease that can cause cartilage, bone damage, and disability [1]. The tight control of RA disease activity by following the treat-to-target (T2T) strategy to reach optimal outcomes is thus recommended [2]. Advances in the treatment of RA such as the use of biological disease-modifying anti-rheumatic drugs (bDMARDs) and targeted synthetic DMARDs (tsDMARDs) have provided better clinical outcomes (including the achievement of clinical remission) and the prevention of joint damage and disability among individuals with RA.

Abatacept is a soluble fusion protein consisting of cytotoxic T lymphocyte-associated antigen-4 (CTLA-4) and the Fc portion of immunoglobulin G1 [3]. It selectively modulates the CD80/CD86:CD28 costimulatory signal required for full T-cell activation [3]. Both clinical trials [4–7] and clinical practice [8] have demonstrated that abatacept is an effective treatment for patients with RA. Abatacept is thus recommended as one of the first-line bDMARDs for RA [9]. Several studies showed that abatacept strongly inhibits radiographic progression in patients with RA [5–8]. CTLA-4 is suggested to be an anti-osteoclastogenic molecule that directly binds osteoclast precursor cells and inhibits their differentiation [10, 11].

Bone damage in RA is characterized by articular erosions, periarticular bone loss, and secondary osteoporosis caused by chronic inflammation [12]. In the bone metabolism of RA, osteoclastogenesis/bone resorption is promoted and bone formation is inhibited. Regarding the underlying mechanisms, important roles are known to be played by the interaction of immune responses mediated by proinflammatory cytokines such as tumor necrosis factor (TNF), interleukin (IL)-6 and IL-17, and bone biomarkers such as receptor activator of nuclear factor kappa-B ligand (RANKL), osteoprotegerin (OPG), dickkopf-1 (Dkk-1), and sclerostin (SOST) [12–15]. Effects of bDMARDs including TNF inhibitors [16, 17] and IL-6 inhibitors [18–20] on bone biomarkers in patients with RA have been reported, but the effect of abatacept on bone biomarkers in patients with RA has not been investigated. Here, we measured the serum levels of five bone biomarkers (Dkk-1, SOST, osteocalcin [OC], osteopontin [OPN], and OPG) using a multiplex bead assay (bone panel) in RA patients treated with abatacept.

Dkk1 and SOST inhibit the Wnt signaling pathway regulating bone formation [12, 14, 15]. OC is an important component of bone matrix protein, synthesized mainly in osteoblasts [12, 21], and it is a biomarker of bone formation. OPG is a soluble decoy RANKL receptor that inhibits RANKL function, and it is highly involved in inflammatory bone resorption by interfering with RANK–RANKL binding [12, 14, 15]. OPN is strongly expressed in bone, where it promotes the adhesion of osteoclasts to the mineralized matrix regulating bone resorption and formation [22]. OPN has also been recognized as an important proinflammatory mediator that participates in pathological processes such as inflammatory reactions, vasospasm formation, and bone damage [23, 24].

To achieve the goal of the T2T strategy [2], the adequate management of disease activity requires a sensitive and accurate assessment of arthritis. Imaging plays an important role in this assessment. Musculoskeletal ultrasound (MSUS) has been widely applied in clinical settings as an imaging modality for patients with rheumatic diseases [25, 26]. Compared to clinical and radiographic examinations, MSUS provides a straightforward and more accurate detection of both inflammation and damage at the joint level [25, 26]. We have conducted a multicenter prospective observational cohort study of patients with active RA who received bDMARD or tsDMARD therapy at 27 participating rheumatology centers in the Kyushu region of Japan since June 2013: the Kyushu Multicenter Rheumatoid Arthritis Ultrasound Prospective Observational Cohort Study (KUDOS) [27–29]. We evaluated the therapeutic efficacy of the bDMARD and tsDMARD treatments based on the patients’ clinical measurements, MSUS findings, and biomarker assessments. A multicenter collaborative study that prospectively evaluates disease activity by using MSUS standardized at a high level is rare, even worldwide.

In the present study, we evaluated the effect of abatacept treatment on bone biomarkers and explored whether bone biomarkers are associated with the therapeutic response in RA patients treated with abatacept, using the KUDOS data.

## Methods

### Patients

This study is part of an ongoing non-randomized multicenter prospective observational cohort study (i.e., the KUDOS study) of patients with RA who received bDMARD or tsDMARD therapy at one of 27 participating rheumatology centers in Japan’s Kyushu region since June 2013 [27–29]. We evaluated the therapeutic efficacy by determining the patients’ clinical disease activity, MSUS score, and serum biomarkers at baseline and at 3, 6, 9, 12, 18, and 24 months starting from the initiation of treatment with a new bDMARD or tsDMARD. The patients’ previous use of a bDMARD or tsDMARD was not restricted. For the present study, we enrolled the 59 consecutive Japanese patients with RA who were treated with abatacept and had continued the abatacept treatment for > 6 months at 10 participating rheumatology centers during the period from December 2013 to March 2016. All enrolled patients were required to satisfy the 1987 American College of Rheumatology (ACR) [30] and/or 2010 ACR/EULAR (European League Against Rheumatism) criteria for RA [31]. Abatacept was administered as recommended by the manufacturers: 125 mg via subcutaneous injection weekly or 500–750 mg via intravenous infusion every 4 weeks.

We excluded patients who were treated with a newly introduced oral bisphosphonate during the study period or were treated with an intravenous bisphosphonate, anti-RANKL antibody, or parathyroid hormone (PTH) agent. Local injection was not performed, and the use of nonsteroidal anti-inflammatory drugs (NSAIDs) was not restricted. This was an exploratory study investigating whether bone biomarkers are associated with the therapeutic response in RA patients treated with abatacept. Given the T2T strategy [2], we considered short-term data to be important from the perspective of predicting therapeutic response, and we thus used 3 and 6 months as the timing of the post-treatment evaluations.

The study is registered with the University Hospital Medical Information Network Clinical Trials Registry (http://www.umin.ac.jp/ctr/, UMIN 000012524) and was approved by the Institutional Review Board of Nagasaki University (approval no. 13102866). All patients gave their signed informed consent to participate in accordance with the Helsinki Declaration.

### Clinical and laboratory assessments

Disease activity was evaluated by each patient’s attending physician (Japan College of Rheumatology [JCR]-certified rheumatologists) and was based on the Disease Activity Score (DAS) 28-joint C-reactive protein (CRP) value and the Simple Disease Activity Index (SDAI) value at baseline and every 3 months after the introduction of abatacept. The treating physicians were different from the MSUS evaluators. The patients’ baseline MSUS scores were evaluated after the decision regarding the introduction of b/tsDMARD therapy.

### Musculoskeletal ultrasound assessment

The MSUS examination of each patient was performed by JCR-certified sonographers. At all of the participating institutions, a trained MSUS expert examined the patient in an environment recommended by the JCR guidelines, considering factors that can affect power Doppler (PD) results, including the room temperature, the last use of an NSAID, and the subject’s hand position. Medium-level to high-level ultrasound machines were used (Toshiba AplioXG and Aplio300, GE Logic series 7 and 8 or Hitachi Ascendus, Avius, Noblus, and Hi Vision Preirus) with high-frequency (12–18.5 MHz) linear transducers. The Doppler parameters were adjusted according to the device used (range of pulse repetition frequency 500–1000 Hz; Doppler frequency 6.1–10.0 MHz). There was no change in MSUS settings during the study.

Twenty-two joints including the metacarpophalangeal (MCP), proximal interphalangeal (PIP), and wrist joints of each patient’s bilateral hands were assessed by MSUS at baseline and at 3 and 6 months of treatment. The 22 joints were scanned on the dorsal aspect. Standardized joint and probe positions were used, based on a guideline published by the JCR. Each instance of grayscale (GS) synovial hypertrophy and the PD signal were both scored semi-quantitatively on a scale from 0 to 3 [32]. The sum of the GS or PD scores was used as the indicator of US disease activity, described as the total GS score or total PD score. The total scores ranged from 0 to 66. We defined a ‘PD responder’ at 6 months as a patient whose change in total PD score (Δtotal PD score) over 6 months was greater than the median change (i.e., a Δtotal PD score less than or equal to − 4) in all patients. We defined a ‘non-PD responder’ at 6 months as a patient whose Δtotal PD score over 6 months was less than the median change (i.e., a Δtotal PD score greater than or equal to − 3) in all patients. We defined PD remission as a total PD score of 0 at 6 months. Interobserver reliability was confirmed in a previous investigation [27].

### Bone biomarker measurements

We measured the concentrations of the following biomarkers by using serum stored on the same day as the patient’s clinical evaluation. Rheumatoid factor (RF) was measured by the latex agglutination turbidimetric immunoassay (LATIA) (LZ test RF, Eiken Chemical Co., Tokyo). Anti-cyclic citrullinated peptide antibody (ACPA) was measured by a chemiluminescent immunoassay (CLEIA) (STACIA MEBLux™ test CCP, Nagoya, Japan). We performed the multiplex bead assays using diluted serum supernatants and a Milliplex MAP Human Bone Panel analyzed with a Bio-Plex® MAGPIX™ Multiplex Reader (Bio-Rad, Hercules, CA) according to the manufacturer’s instructions. The bone biomarkers measurable by the bead panel that have been reported to be associated with RA included Dkk-1, SOST, OC, OPN, and OPG using a multi-suspension array. These bone biomarkers were also measured in 18 healthy age- and gender-matched volunteers (median age 70 years, 67% females). The volunteers were recruited at medical check-ups in the town of Saza, Japan. The protocol was approved by the Nagasaki University Ethics Committee for Humans Subjects (approval no. 14051404).

### Statistical analyses

Statistical analyses were performed using JMP Pro statistical software, ver. 11.0 (SAS, Cary, NC). Quantitative variables are presented as medians and interquartile ranges (IQRs). Categorical variables are presented as percentages. We used the Mann-Whitney U-test for comparisons between independent medians, and we used the Chi-square test for the evaluation of the associations between categorical variables. Correlations were assessed with Spearman’s correlation coefficient. The changes in clinical disease activity indices, MSUS scores, and serum concentrations of bone biomarkers over 6 months were analyzed using the Wilcoxon signed ranks test. We attempted to identify any variables that were independently predictive of the PD responder status at 6 months from the patients’ baseline characteristics by performing a multivariate logistic regression analysis. All variables with a *p*-value < 0.1 in a univariate analysis were used in the multivariate models, but the SDAI and total GS score were excluded as confounding factors of the total PD score. *P*-values < 0.05 were considered significant.

## Results

### Demographic, clinical, and laboratory characteristics of the 59 RA patients

The patient’s characteristics at baseline are summarized in Table 1. The median (IQR) age of the patients was 72 (65–77) years, and the median (IQR) of the RA disease duration was 54 (14–186) months. The medians (IQR) of the DAS28-CRP and SDAI were 4.20 (3.39–5.01) and 20.7 (13.7–30.7), respectively. The medians (IQR) of the total GS and PD scores were 14 (7–22) and 7 (4–15), respectively. Methotrexate (MTX, median dose: 8 mg weekly) and low-dose oral glucocorticoids (median dose: 5 mg daily) were concomitant in 26 (44.1%) and 33 (55.9%) patients, respectively. Twenty-two (37.3%) patients had a history of previous use of a bDMARD.

**Table 1:** Demographic, clinical and laboratory characteristics of the 59 patients with RA

Age, yrs	72 (65–77)
Male, n	16 (27.1)
Disease duration, months	54 (14–186)
csDMARDs use, n	48 (81.4)
MTX use, n	26 (44.1)
Corticosteroid use, n	33 (55.9)
Previous use of bDMARDs	22 (37.3)
Oral bisphosphonate use	21 (35.6)
Positive RF, n	46 (78.0)
Positive ACPA, n	52 (88.1)
Tender joint counts (28), n	5 (3–10)
Swollen joint counts (28), n	5 (2–10)
PtGA, mm	40 (20–70)
EGA, mm	40 (30–53)
CRP, mg/dl	0.64 (0.12–2.14)
MMP-3, ng/ml	124 (66–288)
DAS28-CRP	4.20 (3.39–5.01)
SDAI	20.7 (13.7–30.7)
Total GS score	14 (7–22)
Total PD score	7 (4–15)

The data are median (interquartile range, Q_1–4_–Q_3/4_) or number (percentage). *ACPA* Anti-cyclic citrullinated peptide antibody, *bDMARDs* biologic disease-modifying antirheumatic drugs, *CRP* C-reactive protein, *DAS* Disease Activity Score, *csDMARDs* Conventional synthetic disease-modifying antirheumatic drugs, *EGA* Evaluator’s global assessment, *ESR* Erythrocyte sedimentation rate, *GS* Gray-scale, *MTX* Methotrexate, *PD* Power Doppler, *PtGA* Patient’s global assessment, *RA* Rheumatoid arthritis, *RF* Rheumatoid factor, *SDAI* Simplified Disease Activity Index

### Improvement of clinical and MSUS activities over 6 months

**Fig. 1:**
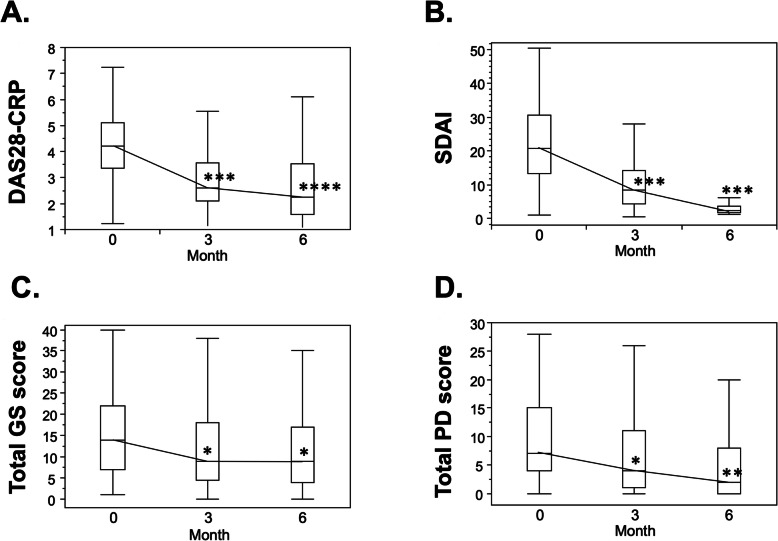
Changes in the clinical disease activity and MSUS scores over the 6-month abatacept treatment period. The DAS28-CRP (**A**), SDAI (**B**), total GS score, and total PD score were significantly improved at 3 and 6 months compared to the baseline. *p < 0.05, **p < 0.001, ***p < 0.0001, Wilcoxon signed ranks test. DAS28-CRP: Disease Activity Score 28-joint C-reactive protein, GS: grayscale, PD: power Doppler, SDAI: Simple Disease Activity Index

Overall, the patients’ DAS28-CRP (Fig. 1A) and SDAI (Fig. 1B) values were significantly improved at 3 and 6 months compared to the baseline (*p* < 0.001, respectively). The median (IQR) of the total GS scores decreased from 14 (7–22) at baseline to 9 (5–18) at 3 months (*p* < 0.05) and 9 (4–17) at 6 months (p < 0.05) (Fig. 1C). In addition, the median (IQR) of the PD scores decreased from 7 (4–15) at baseline to 4 (1–11) at 3 months (p < 0.05) and 2 (0–7) at 6 months (p < 0.001) (Fig. 1D).

### The serum concentrations of the bone biomarkers

The results of our comparison of the serum concentrations of the five bone biomarkers between the healthy volunteers and the RA patients and the changes of the biomarkers over the 6-month period after the introduction of abatacept in the RA patients are summarized in Table 2. Serum OPN was significantly higher (*p* < 0.0001) and serum OC tended to be lower (*p* = 0.099) in the RA patients compared to the healthy volunteers. The other serum bone biomarkers were not significantly different between the RA and healthy groups. Serum OPG was significantly elevated at 6 months after the introduction of abatacept (*p* = 0.016, Table 2, Suppl. Fig. S1), but the other serum bone biomarkers did not change after treatment. The uses of oral corticosteroids and bisphosphonates did not affect the serum concentrations of bone biomarkers or their changes.

**Table 2:** Comparison of serum concentrations of bone biomarkers between healthy controls and RA patients and changes of those over 6 months in the RA patients

	HC	RA					
		Baseline	***p***-value vs. HC^a^	3 months	***p***-value vs. baseline^**b**^	6 months	***p***-value vs. baseline^**b**^
Dkk-1, pg/ml	4362 (2866–5026)	3340 (2573–4243)	0.25	3260 (2519–4282)	0.86	3242 (2689–4251)	0.98
SOST, pg/ml	2509 (1939–3315)	2616 (1983–3279)	0.64	2588 (2171–3506)	0.68	2738 (2127–3465)	0.62
OC, pg/ml	28,492(18700–35,923)	17,109 (11075–35,817)	0.099	18,271 (12754–31,724)	0.52	21,533 (14559–28,715)	0.26
OPG, pg/ml	1036 (896–1184)	951 (768–1387)	0.76	1082 (903–1469)	0.085	1181 (903–1469)	0.016
OPN, pg/ml	9053 (6282–14,337)	22,928 (17305–37,566)	< 0.0001	23,689 (13849–32,156)	0.76	21,818 (13895–33,550)	0.85

The data are median (interquartile range, Q_1–4_–Q_3/4_) or number (percentage)

^a^Comparisons of the serum concentration of bone biomarkers between HC and RA were analyzed using the Mann-Whitney U-test

^b^ The changes in the serum concentration of bone biomarkers from baseline were analyzed using the Wilcoxon signed ranks test. *HC* Healthy control, *PD* Power Doppler, *RA* Rheumatoid arthritis

### The association between the ultrasonographic response and the bone biomarkers

**Fig. 2:**
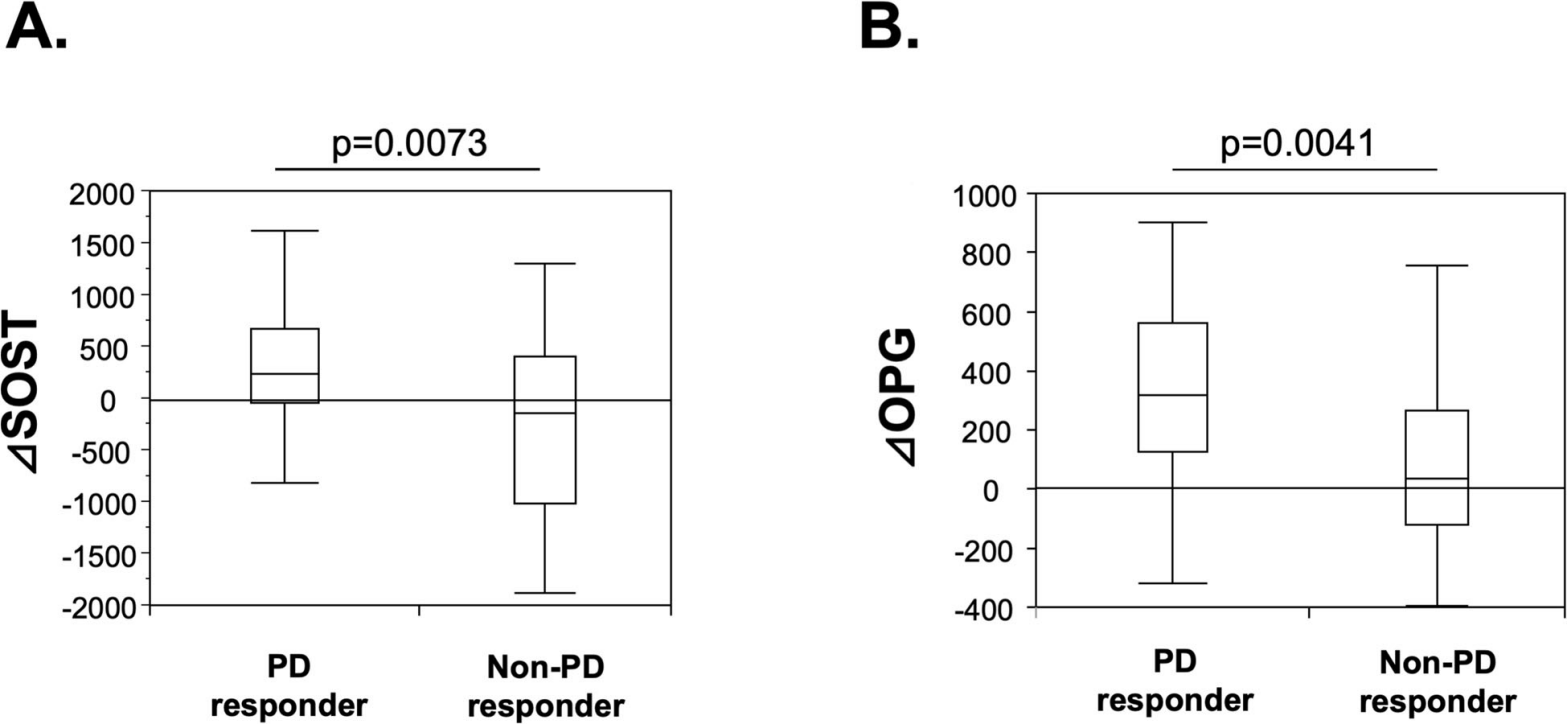
Comparison of ΔSOST and ΔOPG between the PD responders (*n* = 30) and non-PD responders (*n* = 29). Both the ΔSOST and ΔOPG were significantly greater in the PD responders compared to the non-PD responders. Within-group comparisons were made using Mann-Whitney’s U-test. *Horizontal bar:* median, *boxes:* 25th and 75th percentiles, *bars:* 5th and 95th percentiles. OPG: osteoprotegerin, SOST: sclerostin

We compared the changes in the serum concentrations of bone biomarkers (Δ value) between the PD responders and non-PD responders. The ΔSOST and ΔOPG were significantly greater in the PD responders compared to the non-PD responders (*p* = 0.0041 and 0.0073, respectively, Fig. 2). The changes in the other biomarkers were not significantly different between the PD responders and non-PD responders.

The results of our comparison of the patient characteristics at baseline between the PD responders and non-PD responders at 6 months are provided in Table 3. The patient age and disease duration tended to be higher in the PD responders compared to the non-PD responders. RF positivity (*p* = 0.030), the SDAI (*p* = 0.0081), the total GS score (*p* = 0.0005), and the total PD score (*p* < 0.0001) were significantly higher in the PD responders compared to the non-PD responders. The serum level of Dkk-1 was significantly lower in the PD responders compared to the non-PD responders (*p* = 0.026). The serum level of SOST tended to be lower in the PD responders compared to the non-PD responders (*p* = 0.058).

**Table 3:** Comparison of patient characteristics between the PD responders and non-PD responders

	PD responders ***n*** = 30	Non-PD responders ***n*** = 29	***p***-value
Age, yrs	74 (67–78)	70 (62–74)	0.058
Male, n	8 (26.7)	8 (27.6)	1.00
Disease duration, months	92 (26–258)	39 (10–156)	0.071
Positive RF, n	27 (90.0)	19 (65.5)	0.030
Positive ACPA, n	28 (93.3)	24 (82.8)	0.21
SDAI	25.0 (16.6–36.5)	18.2 (12.2–24.5)	0.0081
Total GS score	19 (13–28)	8 (4–15)	0.0005
Total PD score	11 (7–20)	4 (1–7)	< 0.0001
Dkk-1, pg/ml	3040 (2489–3652)	3699 (2676–4477)	0.026
SOST, pg/ml	2428 (1721–3057)	2803 (2137–3916)	0.058
OC, pg/ml	16,588 (9582–28,327)	17,722 (12512–39,103)	0.54
OPG, pg/ml	958 (749–1190)	945 (839–1534)	0.32
OPN, pg/ml	23,035 (17382–40,435)	22,928 (17305–35,962)	0.93

*ACPA* Anti-cyclic citrullinated peptide antibody, *OC* Osteocalcin, *OPG* Osteoprotegrin, *OPN* Osteopontin, *PD* Power Doppler, *RF* Rheumatoid factor, *SDAI* Simplified Disease Activity Index, *SOST* Sclerostin

Based on the results of the univariate analysis, we entered six baseline variables (age, disease duration, RF positivity, total PD score, serum Dkk-1 level, and serum SOST level) into the multivariate regressions (Table 4). The serum level of Dkk-1 at baseline (odds ratio [OR] 0.497, 95% confidence interval [CI] 0.225–0.909, *p* = 0.043) was revealed as the only independent predictor of PD responder status at 6 months.

**Table 4:** Baseline predictors of PD responder at 6 months by multivariate logistic regression analysis

	Comparison	OR	95%CI	***p***-value
Serum Dkk-1	1 ng/ml increase	0.497	0.225–0.909	0.043
Serum SOST	1 ng/ml increase	0.562	0.286–1.001	0.065
Total PD score	1 increase	1.145	0.989–1.372	0.098
RF	positive	3.751	0.684–29.05	0.154
Disease duration	1 yr increase	1.004	0.996–1.011	0.343
Age	1 yr increase	1.009	0.934–1.092	0.811

*PD* Power Doppler, *RF* Rheumatoid factor, *SOST* Sclerostin

We compared the changes in the SDAI and the total PD score between the patients with a low Dkk-1 value (i.e., < the median Dkk-1 value at baseline) and the patients with a high Dkk-1 value (≥ the median Dkk-1 at baseline) (Suppl. Fig. S2) and between the patients with a low SOST value (< the median SOST at baseline) and those with a high SOST value (≥ the median of SOST at baseline) (Suppl. Fig. S3). The SDAI score improved in the patients with a low Dkk-1 value as well as the patients with a high Dkk-1 value and in the patients with a low SOST value as well as the patients with a high SOST value. However, the improvement in the total PD score was better in the patients with low Dkk-1 values compared to those with high Dkk-1 values.

## Discussion

We evaluated the association between the serum concentrations of five bone biomarkers and the therapeutic response to abatacept in RA patients, using the data obtained in our multicenter prospective ultrasound cohort study (KUDOS). In the present study, since abatacept significantly improved the patients’ clinical disease activity as well as their MSUS score over the 6-month treatment, bone destruction was expected to be prevented in this population. Our present analyses revealed that the patients’ serum OPG was significantly elevated at 6 months after the introduction of abatacept. The ΔSOST and ΔOPG were significantly greater in the PD responders compared to the non-PD responders. A multivariate logistic regression analysis demonstrated that the serum Dkk-1 concentration at baseline was an independent predictor of PD responder status.

We observed that both Dkk-1 and SOST (which are inhibitors of the Wnt signaling pathway) were associated with the therapeutic response — especially the ultrasonographic response evaluated by the total PD score. Low serum levels of Dkk-1 at baseline was an independent predictor of the PD responder status at 6 months. Low serum levels of SOST at baseline tended to be associated with the PD responder status at 6 months. Increased serum levels of SOST were significantly correlated with the improvement of disease activity after treatment with abatacept. The Wnt signaling pathway plays a key role in several biological processes such as cellular proliferation and tissue regeneration, and its dysregulation is involved in the pathogenesis of many autoimmune diseases [14]. In RA, Wnt signaling is implicated in systemic and localized bone loss. This process involves proinflammatory cytokines produced by the synovial membrane, which may increase bone resorption but also stimulates soluble antagonists of the canonical Wnt/β-catenin signaling pathway (including DKK-1 and sclerostin) and subsequently inhibits osteoblast proliferation, maturation, and progenitor differentiation [14, 33, 34].

In particular, the pro-inflammatory cytokines TNF and IL-1 induce Dkk-1 and SOST; IL-17 down-regulates the Wnt/β-catenin pathway indirectly, enhancing the production of TNF and IL-1, and IL-6 induces the differentiation of B cells into plasma cells which express Dkk-1 [14, 35, 36]. Moreover, Dkk-1 induces SOST [14]. It has been demonstrated that Dkk-1 promotes synovial angiogenesis, a critical process in the pathogenesis of RA [34]: vascular proliferation occurs during pannus formation in the affected joints [37, 38], during which the synovium becomes locally invasive at the interface with cartilage and bone. The serum level of Dkk-1 has been shown to be higher in patients with RA than in controls and to correlate with bone erosions and inflammation [34]. Increased serum levels of Dkk-1 and SOST may therefore indicate a poor prognosis and resistance to treatment in patients with RA. In the present patient series, the serum levels of Dkk-1 and SOST at baseline were closely associated with the responsiveness of PD activity, which reflects synovial angiogenesis [37, 39] and predicts joint destruction [40].

Regarding the effect of bDMARD therapy on the Wnt signaling in RA, most of the relevant studies were conducted with TNFα inhibitors [34, 41] or an IL-6 receptor (IL-6R) inhibitor, tocilizumab [18, 19], and they showed a decrease in the serum level of Dkk-1 in RA patients undergoing these bDMARD treatments. A reduction in the serum level of Dkk-1 may result from the inhibition of the TNFα- and IL-6-dependent induction of Dkk-1 by TNFα inhibitors or anti-IL-6 monoclonal antibody. On the other hand, abatacept did not reduce the serum level of Dkk-1 in the present RA patients, perhaps because it does not directly inhibit TNFα and IL-6. Aside from the present study, there is no report regarding the effect of abatacept on the Wnt signaling in RA patients [14].

In healthy mice, abatacept promoted bone formation by inducing the Wnt ligand Wnt-10b in a T cell-dependent manner [42], but paradoxically it increased the expression of SOST [41]. Bone formation has been suggested to be moderated by a direct negative feedback loop involving a putative CTLA-4 Ig association with CD80/CD86 on osteoblasts, causing the production of SOST [14, 43]. In the present patient series, an increased serum level of SOST was significantly correlated with the improvement of disease activity after the introduction of abatacept.

We also observed that the serum level of OPG was significantly elevated after the introduction of abatacept, and this elevation was significantly correlated with the improvement of disease activity. The RANK-RANKL system is the major driver of bone destruction in inflammatory arthritis [12, 14, 15]. This system is promoted by proinflammatory cytokines such as TNFα and IL-6 [12, 14, 15]. OPG, a decoy receptor of RANKL, influences bone erosions in RA [15, 44]. A low OPG/RANKL ratio has thus been associated with increased radiographic damage in RA [15, 45]. Wnt signaling is involved in osteoclastogenesis regulation; the canonical Wnt/β-catenin signaling pathway leads to an up-regulation of OPG and a down-regulation of RANKL [14, 35]. TNF inhibitors [14, 46] and an IL-6R inhibitor [14, 18, 19] increased both the expression of OPG and the OPG/RANKL ratio, possibly due to a promotion of Wnt signaling following a decrease in DKK-1. Similarly, abatacept may elevate the serum level of OPG because it promotes Wnt signaling as described above. In addition, the serum levels of OPN among our patients were higher and those of OC tended to be lower compared to the healthy volunteers, as in previous reports [21, 23]. However, we did not observe any effect of abatacept treatment on these bone biomarkers.

Some limitations of our study should be mentioned. The limited sample size (*n* = 59) does not allow for subanalyses of differences due to the patients’ heterogeneous characteristics. Detailed analyses of larger sample sizes may be necessary to verify our present findings. However, our results are valuable as a part of a multicenter collaborative study that prospectively and thoroughly evaluated disease activity using MSUS. Second, we could not evaluate structural changes in joints. In the cohort study, we evaluated the patients’ X-ray images at baseline, 6, 12, 18, and 24 months. We will explore whether bone biomarkers are associated with radiographic progression in RA patients treated with abatacept by using long-term data.

## Conclusions

This is the first study to evaluate the effects of abatacept treatment on bone biomarkers in RA patients and to explore whether bone biomarkers are associated with the patients’ therapeutic response by using the data obtained in the KUDOS study. In particular, the present investigation is unique in that it evaluated the therapeutic response confirmed by MSUS. Abatacept may prevent bone destruction through the promotion of the Wnt signaling pathway. In addition, the measurements of the serum levels of bone biomarkers may be useful for predicting the ultrasonographic response to abatacept.

## Supplementary Information

**Additional file 1: Fig. S1.** Changes in the serum levels of OPG over the 6-month abatacept treatment period. Serum OPG was significantly elevated at 6 months after the introduction of abatacept (Wilcoxon signed ranks test). Horizontal bar, median; boxes, 25th and 75th percentiles; bars, 5th and 95th percentiles. OPG: osteoprotegerin. **Fig. S2.** Comparison of the changes in the SDAI and total PD score between the patients with a low Dkk-1 (*n* = 30) and those with a high Dkk-1 (*n* = 29). Wilcoxon signed ranks test. Horizontal bar, median; boxes, 25th and 75th percentiles; bars, 5th and 95th percentiles. PD: power Doppler, SDAI: Simple Disease Activity Index. **Fig. S3.** Comparison of the changes in the SDAI and total PD score between the patients with a low SOST (*n* = 30) and those with a high SOST (*n* = 29). Wilcoxon signed ranks test. Horizontal bar, median; boxes, 25th and 75th percentiles; bars, 5th and 95th percentiles.

## Data Availability

The datasets used and/or analyzed during the present study are available from the corresponding author on reasonable request.

## Abbreviations

ACPA: Anti-cyclic citrullinated peptide antibody
ACR: American College of Rheumatology
bDMARD: Biologic disease-modifying antirheumatic drug
CRP: C-reactive protein
CTLA-4: Cytotoxic T lymphocyte-associated antigen-4
DAS: Disease Activity Score
Dkk-1: Dickkopf-1
DMARD: Disease-modifying antirheumatic drug
EULAR: European League Against Rheumatism
GS: Grayscale
IQR: Interquartile range
JCR: Japan College of Rheumatology
KUDOS: Kyushu Multicenter Rheumatoid Arthritis Ultrasound Prospective Observational Cohort Study
MCP: Metacarpophalangeal
MSUS: Musculoskeletal ultrasound
NSAID: Nonsteroidal anti-inflammatory drug
OC: Osteocalcin
OPG: Osteoprotegerin
OPN: Osteopontin
PD: Power Doppler
PIP: Proximal interphalangeal
RA: Rheumatoid arthritis
SDAI: Simple Disease Activity Index
SOST: Sclerostin
T2T: Treat-to-target
tsDMARD: Targeted synthetic DMARD
