# Supplementary material for: Association between serum bone biomarker levels and therapeutic response to abatacept in patients with rheumatoid arthritis (RA): a multicenter, prospective, and observational RA ultrasound cohort study in Japan
Source: BMC Musculoskelet Disord. 2021 Jun 1;22:506. doi: 10.1186/s12891-021-04392-5 (PMC8171043; doi:10.1186/s12891-021-04392-5)
Supplement: Supplementary file 1 — Additional file 1: Fig. S1. Changes in the serum levels of OPG over the 6-month abatacept treatment period. Serum OPG was significantly elevated at 6 months after the introduction of abatacept (Wilcoxon signed ranks test). Horizontal bar, median; boxes, 25th and 75th percentiles; bars, 5th and 95th percentiles. OPG: osteoprotegerin. Fig. S2. Comparison of the changes in the SDAI and total PD score between the patients with a low Dkk-1 (n = 30) and those with a high Dkk-1 (n = 29). Wilcoxon signed ranks test. Horizontal bar, median; boxes, 25th and 75th percentiles; bars, 5th and 95th percentiles. PD: power Doppler, SDAI: Simple Disease Activity Index. Fig. S3. Comparison of the changes in the SDAI and total PD score between the patients with a low SOST (n = 30) and those with a high SOST (n = 29). Wilcoxon signed ranks test. Horizontal bar, median; boxes, 25th and 75th percentiles; bars, 5th and 95th percentiles. [file 12891_2021_4392_MOESM1_ESM.docx]

Supplementary Figure S1.

**Suppl. Fig. S1.** Changes in the serum levels of OPG over the 6-month abatacept treatment period. Serum OPG was significantly elevated at 6 months after the introduction of abatacept (Wilcoxon signed ranks test). Horizontal bar, median; boxes, 25th and 75th percentiles; bars, 5th and 95th percentiles. OPG: osteoprotegerin.

Supplementary Figure S2

**Suppl. Fig. S2.** Comparison of the changes in the SDAI and total PD score between the patients with a low Dkk-1 (n=30) and those with a high Dkk-1 (n=29). Wilcoxon signed ranks test. Horizontal bar, median; boxes, 25th and 75th percentiles; bars, 5th and 95th percentiles. PD: power Doppler, SDAI: Simple Disease Activity Index.

Supplementary Figure S3

**Suppl. Fig. S3.** Comparison of the changes in the SDAI and total PD score between the patients with a low SOST (n=30) and those with a high SOST (n=29). Wilcoxon signed ranks test. Horizontal bar, median; boxes, 25th and 75th percentiles; bars, 5th and 95th percentiles.
